# An international cross-sectional study of dementia researchers’ own perspectives on patient and public involvement

**DOI:** 10.1038/s41598-025-33955-y

**Published:** 2025-12-29

**Authors:** Peter Fusdahl, Daniel Camilo Hernandez, Jonathan Patricio Baldera, Arvid Rongve, Ara Khachaturian, Dag Aarsland, Ingelin Testad, Miguel Germán Borda

**Affiliations:** 1 Centre for Age-Related Medicine (SESAM), Stavanger University Hospital, Stavanger, Norway; 2 Department of Clinical Science (K2), Faculty of Medicine, University of Bergen, Bergen, Norway; 3 Semillero de Neurociencias y Envejecimiento, Ageing Institute, Medical School, Pontificia Universidad Javeriana, Bogotá, Colombia; 4 Instituto de Investigación en Salud, Facultad de Ciencias de la Salud, Universidad Autónoma de Santo Domingo, Santo Domingo, Dominican Republic; 5 Department of Research and Innovation, Haugesund Hospital, Haugesund, Norway; 6 Campaign to Prevent Alzheimer’s Disease, Potomac, MD USA; 7 Brain Watch Coalition of the Campaign to Prevent Alzheimer’s Disease, Potomac, MD USA; 8 International Neurodegenerative Disease Research Center, International Neurodegenerative Disorders Research Center, Prague, Czech Republic; 9 University of Las Vegas, Nevada, National Supercomputing Institute & Dedicated Research Network, Las Vegas, NV USA; 10 Centre for Healthy Brain Ageing, Institute of Psychiatry, Psychology, and Neuroscience, King’s College London, London, UK; 11 Department of Neurology, Clínica Universidad de Navarra, Pamplona, Spain; 12 Centro de Investigación en Ciencias de la Salud (CICSA), FCS, Universidad Anáhuac México Campus Norte, Huixquilucan, Edo. de México, Mexico City, Mexico

**Keywords:** PPI, Dementia, Public engagement, Research quality, Regional disparities, Medical research, Geriatrics, Health policy

## Abstract

Patient and Public Involvement (PPI) can enhance the quality and relevance of health research. However, its implementation remains uneven across regions and research fields. This study explores how dementia and aging researchers perceive PPI’s impact on their work. A cross-sectional survey was distributed to 392 researchers in Europe, Latin America, the USA, and Canada. Following multiple expert reviews, native speaker input, and pilot testing, the final survey was administered. Quantitative data were analyzed using descriptive statistics, group comparisons, and logistic regression models. Of 392 questionnaires, 91 were returned (23.2% response rate). Researchers in Europe, the USA, and Canada reported greater familiarity with PPI than those in Latin America. Notably, 45.1% of respondents selected “I prefer not to answer” when asked about PPI’s role in their research; this was more common among Latin American participants, who also reported lower PPI knowledge. Logistic regression revealed that familiarity with PPI was associated with having more research experience, submitting more grant applications, better access to PPI groups, and using PPI to aid recruitment. These findings point to gaps in the awareness and application of PPI in aging and dementia research, with many researchers expressing uncertainty about its value. Regional disparities underscore the need for consistent, standardized approaches to PPI. Future efforts should focus on closing the gap between theoretical familiarity and active implementation to improve research quality and public engagement, particularly in under-resourced settings.

**Supplementary Information:**

The online version contains supplementary material available at 10.1038/s41598-025-33955-y.

## Introduction

Increasingly, funding bodies expect researchers to engage with interest-holders beyond academia^1^. In Europe, the European Commission introduced Responsible Research and Innovation (RRI) to foster the design of inclusive and sustainable research that benefits society^2,3^. While not legally mandated, many funding bodies, including the European Union, now require researchers to demonstrate how they will involve patients and the public in their research proposals^4,5^. This emphasis on PPI stems from a growing recognition that such engagement enhances the relevance, quality, and impact of research in society. For instance, the Horizon Europe program explicitly encourages co-creation with citizens to ensure that research aligns with societal needs and expectations.

The anticipatory importance of PPI lies in its ability to improve research by incorporating the lived experiences of patients and the general public, leading to more meaningful and applicable results^6^. Furthermore, involving non-researchers in the research process can increase public trust and accountability, which is particularly important in health-related research. Nevertheless, the implementation of PPI has been limited, particularly in the development of clinical practice guidelines (CPGs)^7^, despite the international standards emphasizing its importance for producing high-quality, evidence-based guidelines^8^. The evidence supporting the effectiveness of PPI is also limited^9^, and findings made are often inconsistent or inconclusive^10^. A recent study suggested that incorporating user involvement have the potential to positively impact the research process in the planning and execution phases by providing unique insights and fostering greater engagement with research outcomes^11,12^. However, evidence of PPI’s effectiveness in the dissemination and utilization of research results, particularly in regions like Latin America, remains sparse and underexplored.

The involvement of multiple interest-holders, whose priorities shift with political and societal changes, further complicates this context^13^. Additionally, the lack of standardized criteria for defining public health needs^14,15^ or measuring research returns makes it difficult to align scientific knowledge with policymakers’ funding allocations^15–17^.

Earlier PPI studies focus on implementation and barriers to involving e.g. persons with lived experience, lay persons and patients, in the planning and execution phases of medical research^18,19^. The arguments for such PPI can be practical and normative. PPI can help informant recruiting/retention and research quality^20^, identify research gaps not apparent to academic researchers alone and encourage the development of interventions that are more likely to be effective and accepted by the public^21^, and where normative arguments can be that democratic participation in research and reduce research discrimination^22^. Thus, it can be argued that well-executed engagement with patients and the public in research not only enhances transparency but ensures that research reflects the priorities and concerns of those directly affected^23^, PPI supports alignment of research outcomes with societal needs and relevance to real-world issues. PPI members contribute throughout research by shaping priorities, refining study design, supporting recruitment, guiding data collection, and aiding dissemination. They offer unique lived experiences that improve research relevance and ethics, ensuring studies better reflect patient needs. For example, in PREDICTOM, a multi-national Alzheimer’s research project to identify scalable biosignatures for early and accurate diagnosis of Alzheimer’s disease and personalized preventive interventions, PPI groups from EU, Norway and Spain engage from project planning to feedback on materials and results, influencing decisions at each phase^24^. The Norwegian WiseAge group at exemplifies successful integration by appointing user representatives who partner with researchers over multiple dementia research projects^25^. The multi-stage, collaborative involvement model illustrates how PPI enhances research quality and impact.

The research literature describes potential benefits of PPI but measuring evidential impact of PPI is complex and vague, and the implementation of PPI is fragmented and non-standardized^26^. The PPI barriers are extensive, from planning through execution and evaluation. These barriers include, but are not limited to, understanding of PPI roles and expectations, communication, time and resources, training, power dynamics, institutional commitment, requitement and representativeness, theoretical foundation, equality and diversity^27^.

Notably, how medical researchers perceive the expectation and requirements of engaging and executing these PPI activities is, however, not well documented. Dementia is a leading global public health and cost burden, yet remains underfunded and without an effective cure or prevention^28^. Dementia research is scarce in low- and middle-income countries compared to high-income countries^29^. Dementia research includes research from biomedicine, medical interventions to health care, across different medical research areas, e.g. aging medicine, geriatrics, geriatric psychiatry and dementia. Accessing these researchers required access to larger established research networks, with more participants and more diverse participation^30^. This study aims to fill this PPI knowledge gap for medical researchers in the fields of aging, geriatrics, geriatric psychiatry and dementia reporting to have their main working affiliation in Europe, USA/Canada and Latin America, with high- and middle-income economies^31^.

## Methods

### Study design

This study employed a cross-sectional design using an anonymous, web-based survey to collect quantitative data from researchers in the fields of aging, geriatrics, geriatric psychiatry, and dementia with main working affiliation in Europe, Latin America, the USA, and Canada. The survey was designed and validated through a multi-step process involving expert review, native speaker input, and pilot testing prior to distribution. The study design, the survey design/validation, and the analysis/interpretation of the data were executed by the authors, who are experienced dementia researchers.

### Participants

The participants of this study were existing members of nine national medical associations and research networks across Europe, Latin America, Canada and the United States, all specializing in fields related to aging, geriatrics, geriatric psychiatry and dementia. The questionnaire was not used as part of any recruitment strategy. A questionnaire was distributed to 392 active clinical researchers.

### Data source and survey platform

The data was obtained through an anonymous cross-sectional survey that included closed and open-ended questions to provide a comprehensive understanding, with quantitative data and qualitative data offering deeper insights to explain underlying trends.

The survey included both quantitative and qualitative questions. Quantitative questions were included to promote closed-ended demographic clarity, to aid a timely survey response time, and to standardize the range of answers. Qualitative questions were included to allow respondents to allow answers outside the closed-ended quantitative options.

The qualitative data provided by the participants was limited and non-descriptive, limiting the viability of meaningful analysis. Therefore, in this paper we analyzed only quantitative data. To ensure anonymity and enhance comparability, questions were dichotomized as follows:

- “Yes vs. No” for binary decisions,
- “Very good/Good vs. Not good” to assess quality or satisfaction, and.
- “Always/most of the time/Sometimes vs. No” or “Always/most of the time/Sometimes vs. Never/Not relevant” for evaluating the frequency of specific experiences or usage.

This structured approach categorized responses clearly, enabling efficient cross-tabulation analyses and facilitating the use of logistic regression models.

The survey was executed on the web-based *Nettskjema.no* platform, which is specifically designed to gather sensitive data securely and confidentially, with built in functionality for conducting anonymous surveys^32^.

The survey data used is anonymous, contains no personal data and does not include any special categories of personal data, ref Art. 9 GDPR (General Data Protection Regulation) and such anonymous data do not require any ethics approval or specific consent.

### Validation

The questionnaire used in this study was developed through a multi-step process to evaluate its validity in providing meaningful data, ensuring result repeatability, and its suitability for the intended purpose^33^.

Dichotomized responses were selected to allow cross-tabulation and logistic regression, but this format may reduce nuance. To ensure that the questionnaire still captured realistic perspectives, extensive validity testing was undertaken. Face and content validity were reviewed by seven professors in dementia, public health, and social sciences, while cultural and language validity were assessed through two international focus groups and rigorous translation–back-translation. These procedures confirmed that, despite dichotomization, the items adequately reflected researchers’ views on PPI, representing a pragmatic balance between analytic feasibility and validity.

The validation process included six revisions before the final versions were written in English and Spanish.

The survey was designed so that all the questions must be answered before submitting. The respondents were allowed to answer, “I don’t know” or “I prefer not to answer”. The option to answer “Other” were included for applicable multiple-choice questions. Sex and age were not included in the descriptive questions to ensure anonymity.

The questionnaire had 72 questions including skip questions to previous answers. The questions included multiple-choice options, follow-up questions (if the standard questions did not fit), numerical fill-in boxes and short answers. See the complete questionnaire in supplemental material 2. Numerical fill-in boxes were used for questions where appropriate with respect to the type of answer and potential direct/indirect identification of the respondent.

The development and validation of the questionnaire began with an initial review aimed at assessing how effectively the content would facilitate data collection. Despite a comprehensive literature search, no relevant existing questionnaires were found. The first draft underwent revisions by four professors specializing in age-related and dementia research, all with extensive experience in public health management and research funding. A subsequent revision was conducted by three professors from social sciences and business management.

Research networks in Europe, USA/Canada and Latin-America were selected for the recipients of the survey. A key selection criterion of the regions was to be able to ensure appropriate validation of international cultural context and linguistic appropriateness of the survey, in addition to, a viable access to a sufficient number of potential respondents in each region needed to be secured. Dementia research is international, and the respondents of the survey answered which global regions they did most of their research work, and in which other regions they had research collaborations. These regions included Latin-America, Europe, USA/Canada, Asia, Australia and “Other regions” (free text for respondents). The third version questionnaire was reviewed by two focus groups, with dementia researchers fluent in English or Spanish, with experience from working across Europe, the United States, and Latin America. Each focus group consisted of four PhD candidates and postdoctoral researchers in dementia studies. The fourth version was translated by a native Spanish-speaking expert in dementia and age-related research, with another independent dementia researcher providing a back-translation to English to ensure accuracy. This process led to creating a fifth version, which was compared with the original and back-translated texts.

Ethical considerations were addressed when the Data Protection Officer at Stavanger University Hospital reviewed this fifth version to ensure respondent anonymity, resulting in a sixth revision. The sixth version was then pilot-tested with 100 English-speaking clinical researchers and 38 Spanish-speaking clinical researchers from Europe, Latin America, and the United States, all working with dementia and age-related research. This pilot test revealed two technical issues related to skip questions, which were corrected before finalizing the survey. The pilot respondents and pilot data were excluded from the final respondent group and the data used in this study.

In terms of validity, face, and content validity were established through expert reviews and focus group feedback. Internal, language and cultural validity were ensured via bilingual development and input from a multicultural perspective. External validity was enhanced through the diverse participant pool in the pilot test. Finally, construct validity did not apply to this survey due to its exploratory nature within a field that lacks well-established psychometric measures^34^.

### Statistical analysis

First, an exploratory analysis of the variables included in the study was performed. Then, descriptive data analysis was conducted by estimating percentages for categorical variables and presenting means with standard deviations, medians with interquartile ranges, and minimum and maximum values for quantitative variables.

The anticipatory differences in regional structural constraints and cultural differences^28,29,31^, a group comparison analysis was performed to assess differences by region (Latin America and the Caribbean vs. Europe/ USA/ Canada) and researchers’ familiarity with Patient and Public Involvement (Very familiar vs. Little familiar or not familiar) concerning the variables included in the study. Pearson’s chi-square test was used for categorical variables, the Wilcoxon rank-sum test was applied for numeric variables due to data asymmetry identified in the exploratory analysis, and the t-test was used for proportions.

Finally, logistic regression models were fitted to explore the association between researchers’ familiarity with PPI and region as dependent variables, and the variables included in the study as exposure variables. PPI’s models were adjusted for region due to the differences observed in variables related to research production. Model diagnostics were conducted by verifying the necessary assumptions and detecting influential outliers using Cook’s distance. Some observations identified as influential outliers were removed from the models.

Additionally, a visual analysis was performed by plotting odds ratio graphs to illustrate the associations found. A significance level of 0.05 was used for hypothesis testing and adjustments for multiple comparisons were made using the Benjamini & Hochberg method^35^. All statistical analyses were conducted using R software version 4.3.1.

## Results

Ninety-one survey questionnaires were received from the respondents, resulting in a response rate of 23.2%.

Table 1A presents a comparative analysis of descriptive variables by region, focusing on “Latin America and the Caribbean” (*N* = 30) and “Europe/USA/Canada” (*N* = 61). Among the 91 respondents, 40.7% were medical specialists, and 49.5% held PhDs, with significant regional differences (*p* = 0.011). PhDs were more common in “Europe/USA/Canada” (59%) compared to “Latin America and the Caribbean” (30%), where medical specialists were more predominant (60% vs. 31%).

Most respondents (90.1%) conducted clinical research, with no significant associations between regions (*p* = 0.727). Additional demographic details, collaboration patterns, employment differences between public and private sectors, and university or hospital affiliations are shown in Table 1.

**Table 1:** Characteristics of the sample differenced by region.

Variables	Overall	Region for primary working area		*P* value
		Europe/ USA/ Canada	Latin America and Caribbean	
	(*n* = 91)	(*n* = 61)	(*n* = 30)	
Highest level of formal education, (%)				
Graduate Medical school	4 (4.4%)	4 (6.6%)	0 (0%)	**0**.**011**
Medical Specialist	37 (40.7%)	19 (31.1%)	18 (60.0%)	
PhD	45 (49.5%)	36 (59.0%)	9 (30.0%)	
Other	5 (5.5%)	2 (3.3%)	3 (10.0%)	
Main research discipline, (%)				
Clinical	82 (90.1%)	54 (88.5%)	28 (93.3%)	0.727
Basic science	9 (9.9%)	7 (11.5%)	2 (6.7%)	
Researchers collaborated from, (%)				
Latin America and Caribbean	39 (42.9%)	11 (18.0%)	28 (93.3%)	**< 0.001**
Europe	63 (69.2%)	52 (85.2%)	11 (36.7%)	**< 0.001**
USA/Canada	45 (49.5%)	38 (62.3%)	7 (23.3%)	**< 0.001**
Australia	17 (18.7%)	17 (27.9%)	0 (0%)	**< 0.001**
Asia	16 (17.6%)	16 (26.2%)	0 (0%)	**< 0.001**
Other regions	5 (5.5%)	4 (6.6%)	1 (3.3%)	0.487
Respondent employer(s), (%)				
University/College	46 (50.5%)	30 (49.2%)	16 (53.3%)	0.714
University Hospital	58 (63.7%)	45 (73.8%)	13 (43.3%)	**0**.**007**
Public research center	8 (8.8%)	4 (6.6%)	4 (13.3%)	0.343
Private company	6 (6.6%)	1 (1.6%)	5 (16.7%)	**0**.**042**

*Bivariate models between regions and according to PPI familiarity can be found in the Supplementary Table S1 and S2.

Table 2 shows that, overall, two of three (67.0%) respondents had little or no familiarity with PPI. The difference between the regions were, however, significant (*p* = 0.011), as a 86.7% of the respondents from “Latin America and Caribbean” had little or no familiarity, while 57.4% of Europe/USA/Canada respondents answered the same.

**Table 2:** Respondents’ familiarly with PPI.

Variable	Overall	Europe/USA/Canada	Latin America and Caribbean	*P* value
	(*n* = 91)	(*n* = 61)	(*n* = 30)	
Are you familiar with Patient and Public Involvement (PPI) in research?				
Little familiar or not familiar	61 (67.0%)	35 (57.4%)	26 (86.7%)	0.011
Very familiar	30 (33.0%)	26 (42.6%)	4 (13.3%)	

**Table 3:** Differences in “I prefer not to answer” to PPI-related questions by region and familiarity (Dichotomized).

Variables	Overall	USA/Europe/Canada	Latin America and Caribbean	*P* value	Little familiar or Not familiar	Very familiar	*P* value
	(*n* = 91)	(*n* = 61)	(*n* = 30)		(*n* = 61)	(*n* = 30)	
Does your research institution have groups of people that are available for PPI in your research?	7 (7.7%)	4 (6.6%)	3 (10.0%)	0,594	7 (11.5%)	0 (0%)	0.007
How do you think PPI works in the recruitment of participants for research and clinical trials?	41 (45.1%)	22 (36.1%)	19 (63.3%)	0,015	37 (60.7%)	4 (13.3%)	< 0.001
Does PPI improve the quality of your research?	38 (41.8%)	20 (32.8%)	18 (60.0%)	0,016	37 (60.7%)	1 (3.3%)	< 0.001
Do PPI reduce and/or harm the quality of your research?	34 (37.4%)	17 (27.9%)	17 (56.7%)	0,011	32 (52.5%)	2 (6.7%)	< 0.001
How often do you include PPI in your more recent research projects and/or articles?	25 (27.5%)	10 (16.4%)	15 (50.0%)	0,002	24 (39.3%)	1 (3.3%)	< 0.001

**Fig. 1:**
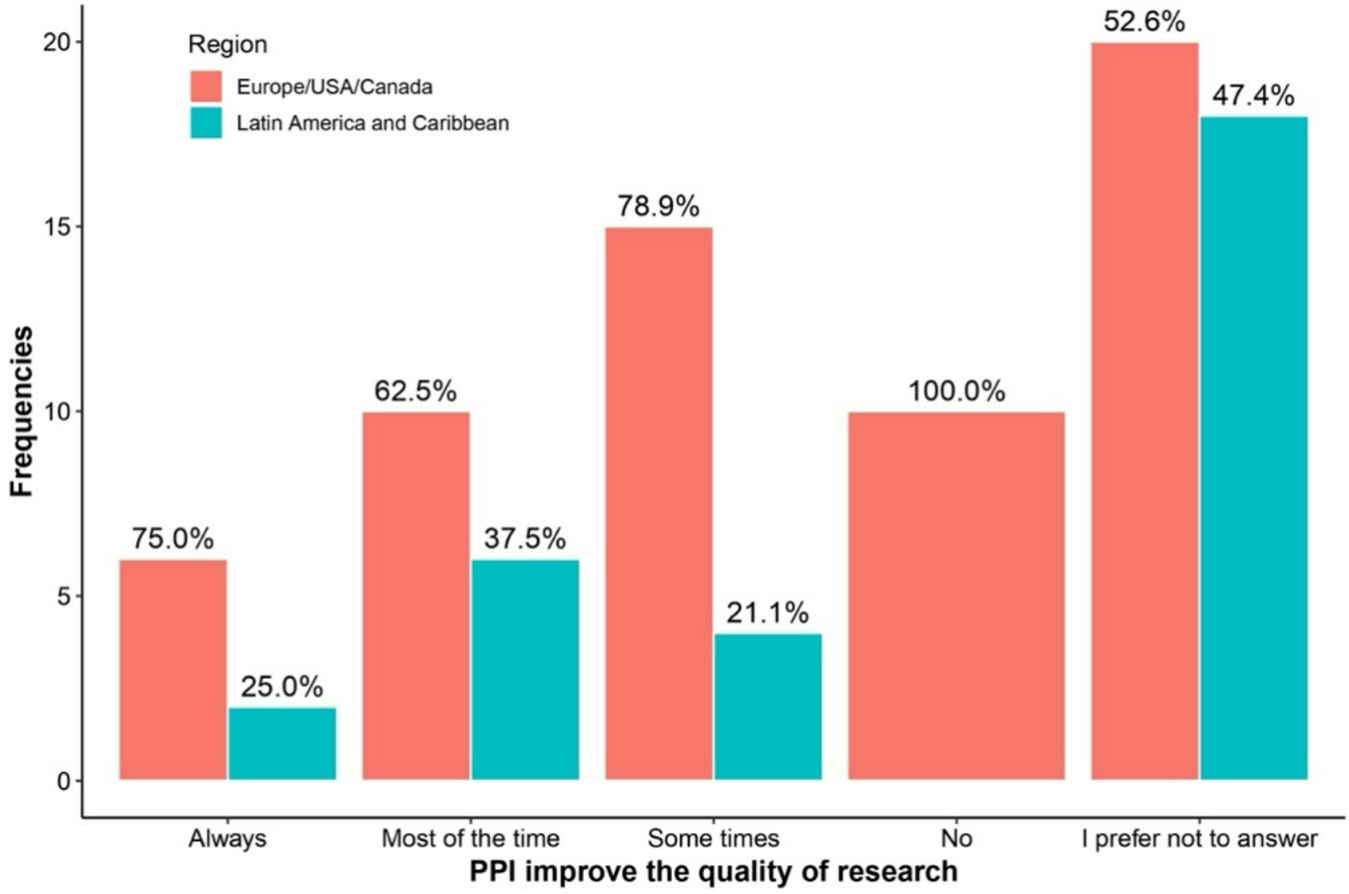
Regional differences on the perceptions of PPI in: Quality of research.

**Fig. 2:**
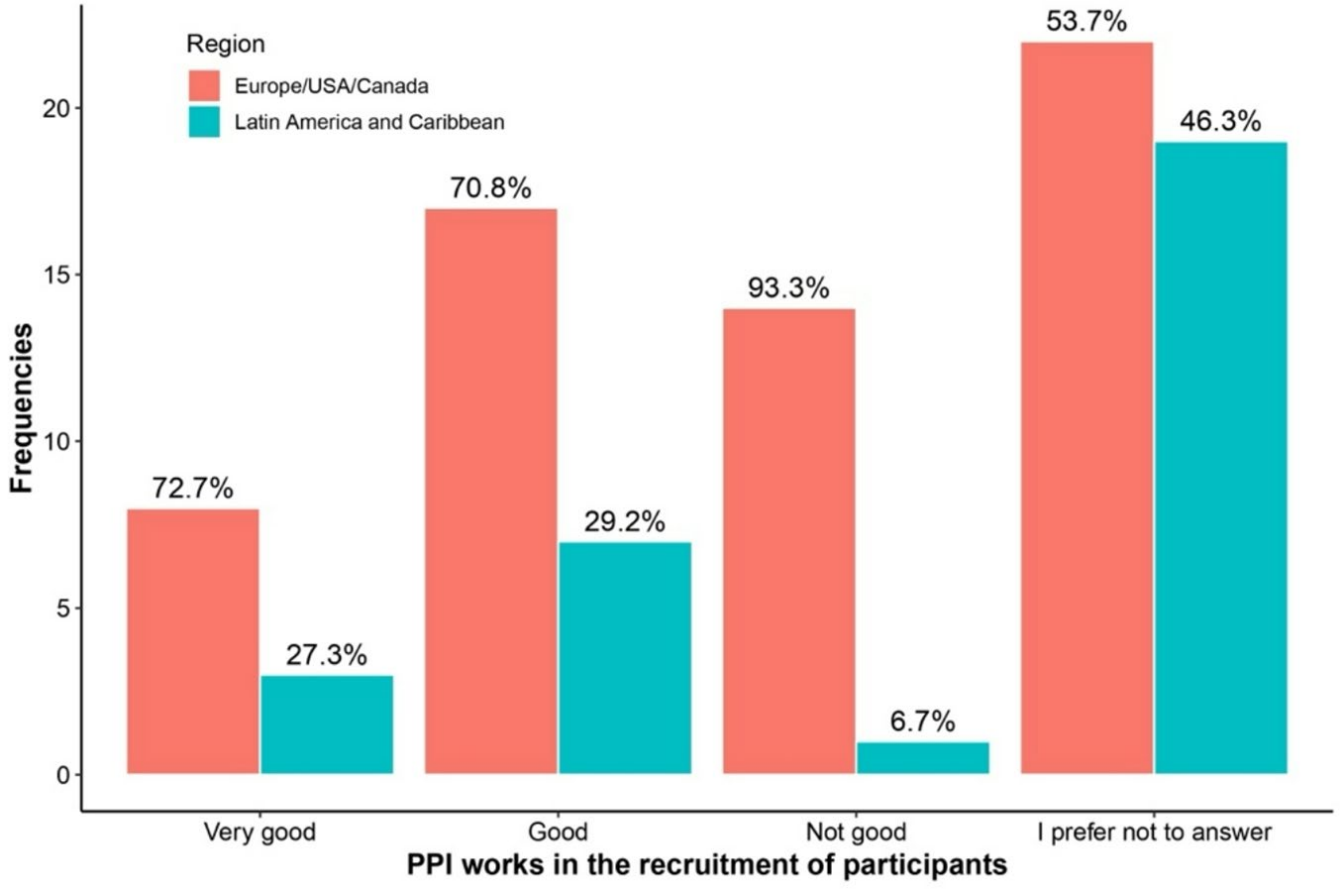
Regional differences on the perceptions of PPI in: Recruitment of participants.

**Fig. 3:**
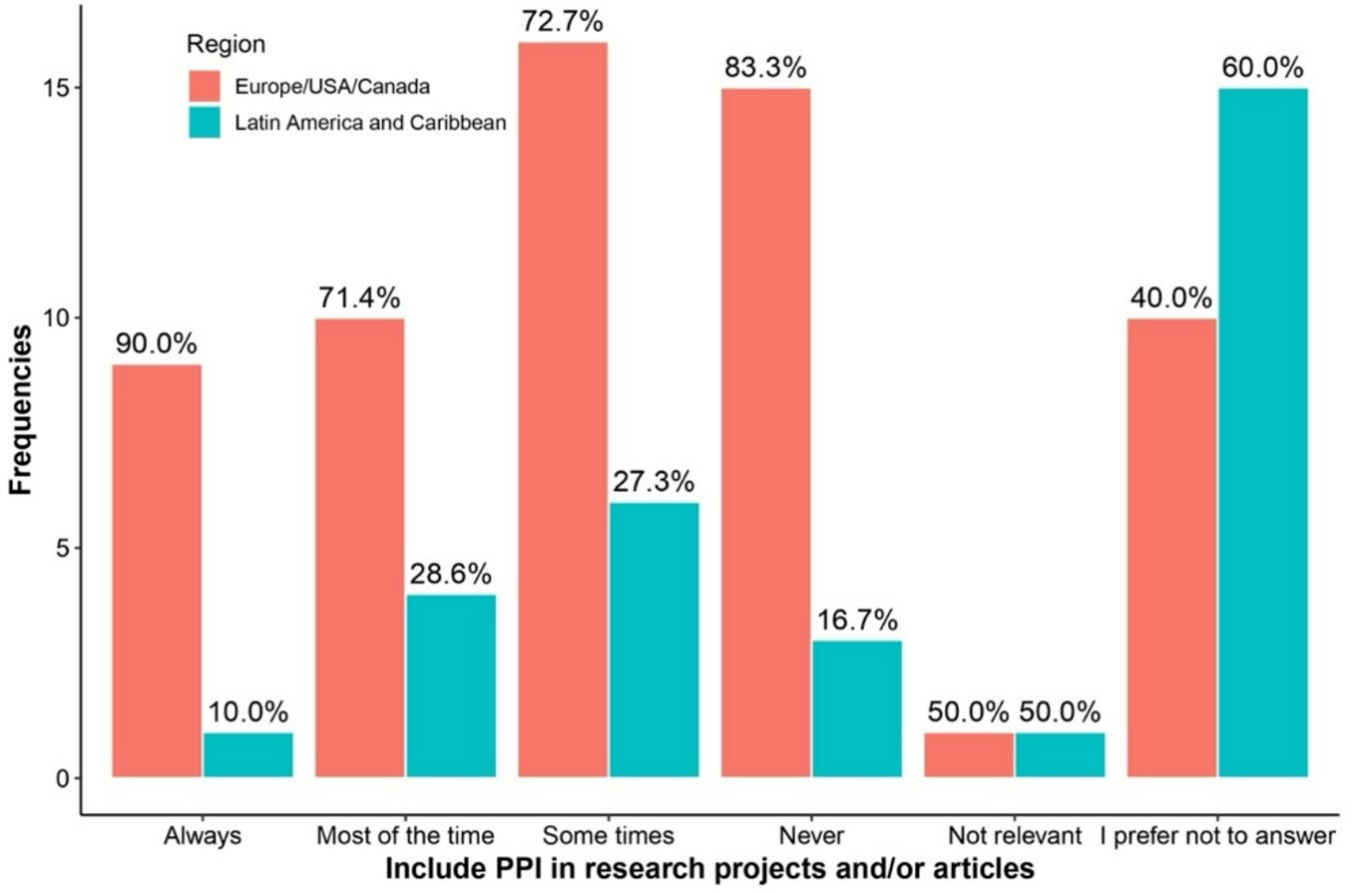
Regional differences on the perceptions of PPI in: Inclusion in research.

**Fig. 4:**
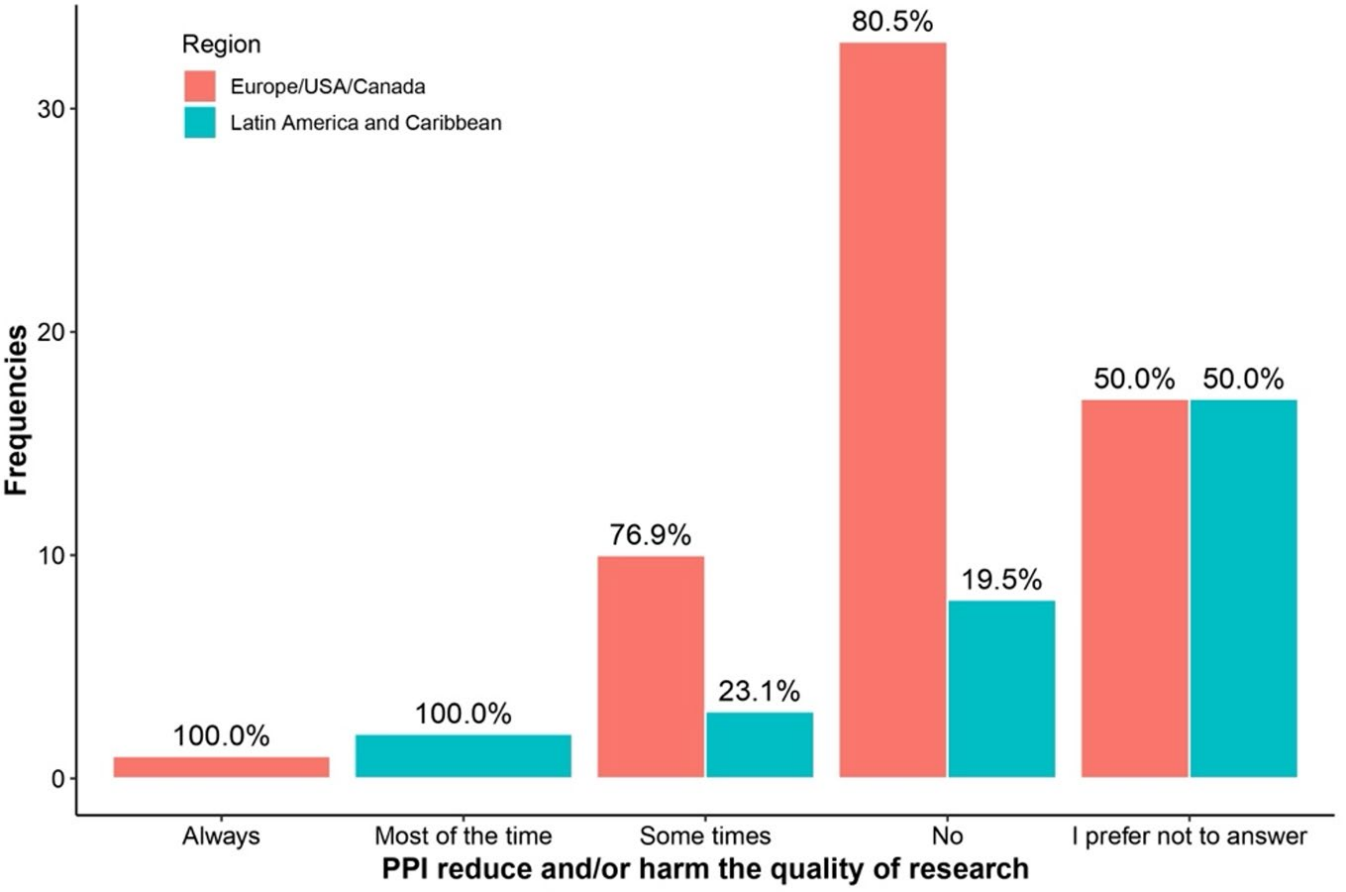
Regional differences on the perceptions of PPI in: Harm to research.

**Fig. 5:**
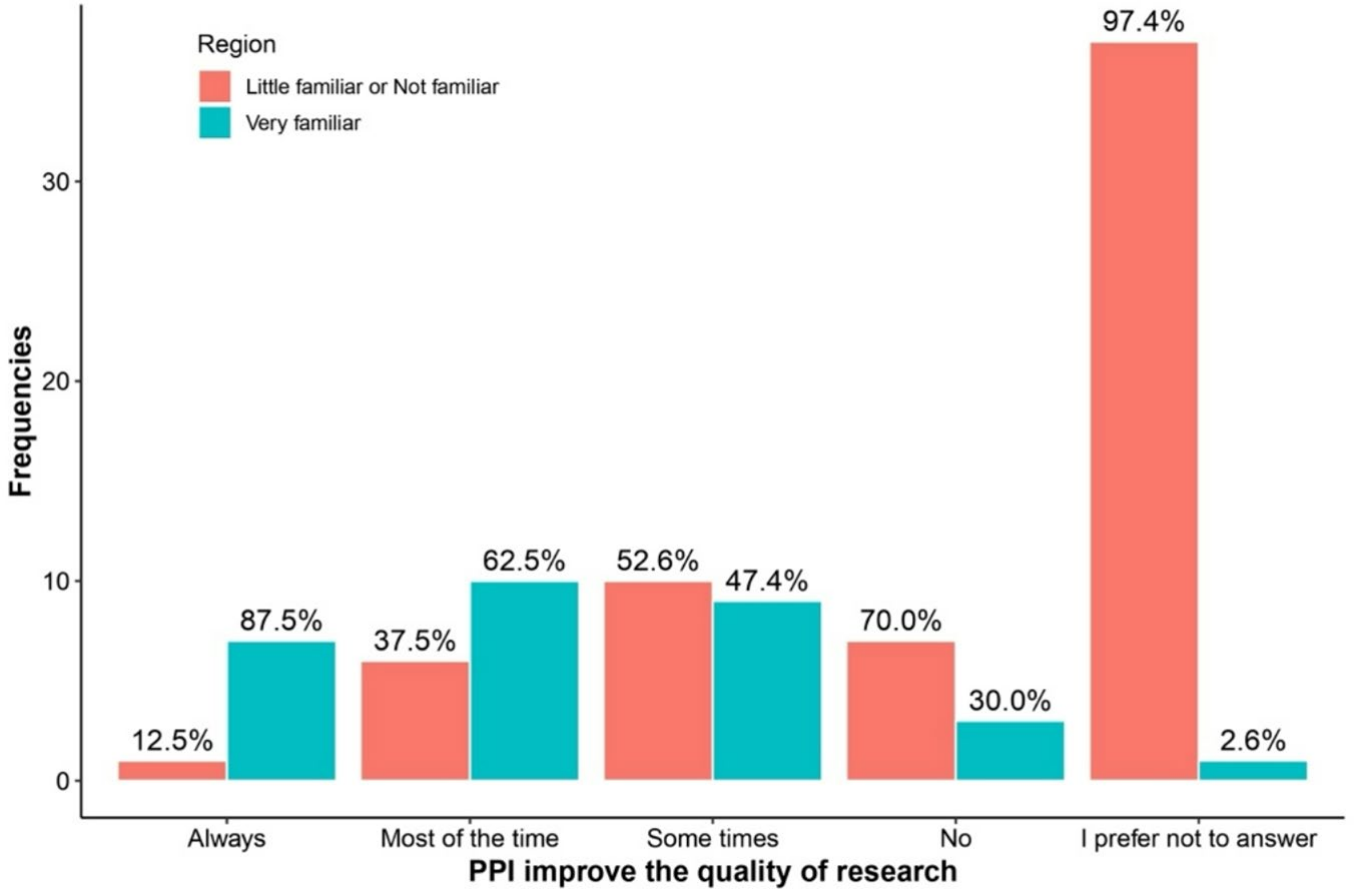
Knowledge of PPI and differences on the perception of PPI with respect to: Quality of research.

**Fig. 6:**
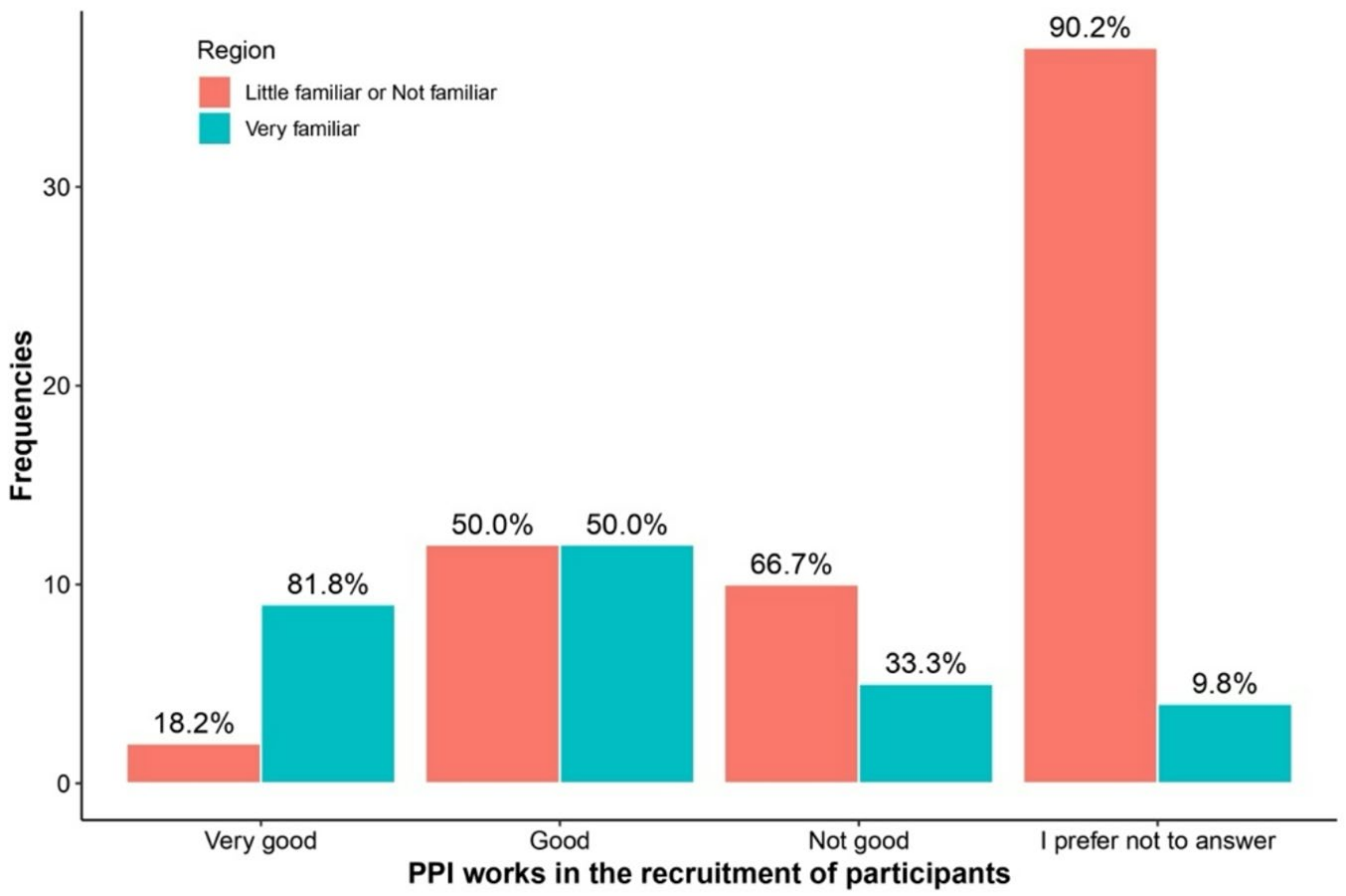
Knowledge of PPI and differences on the perception of PPI with respect to: Recruitment of participants.

**Fig. 7:**
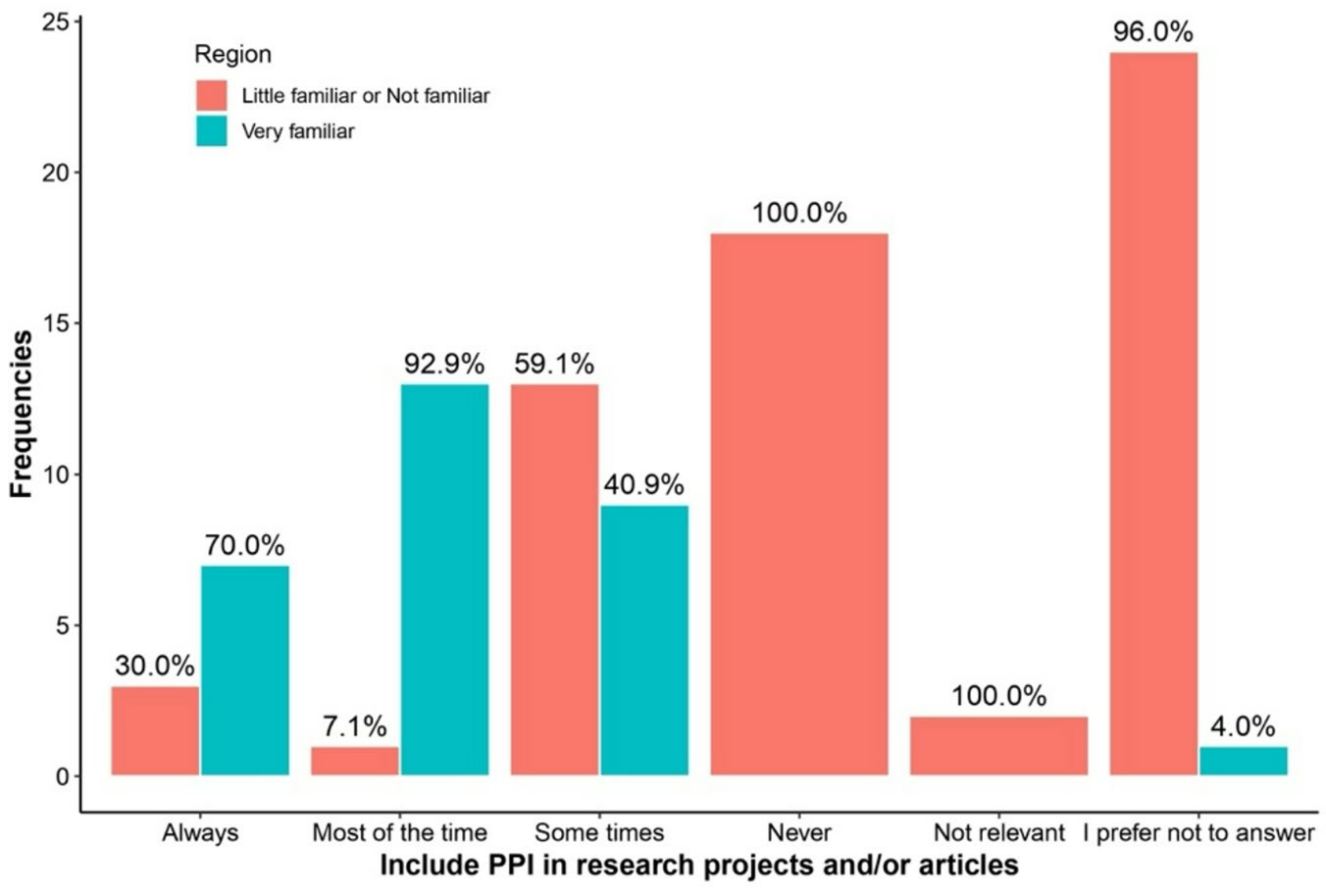
Knowledge of PPI and differences on the perception of PPI with respect to: Inclusion in research.

**Fig. 8:**
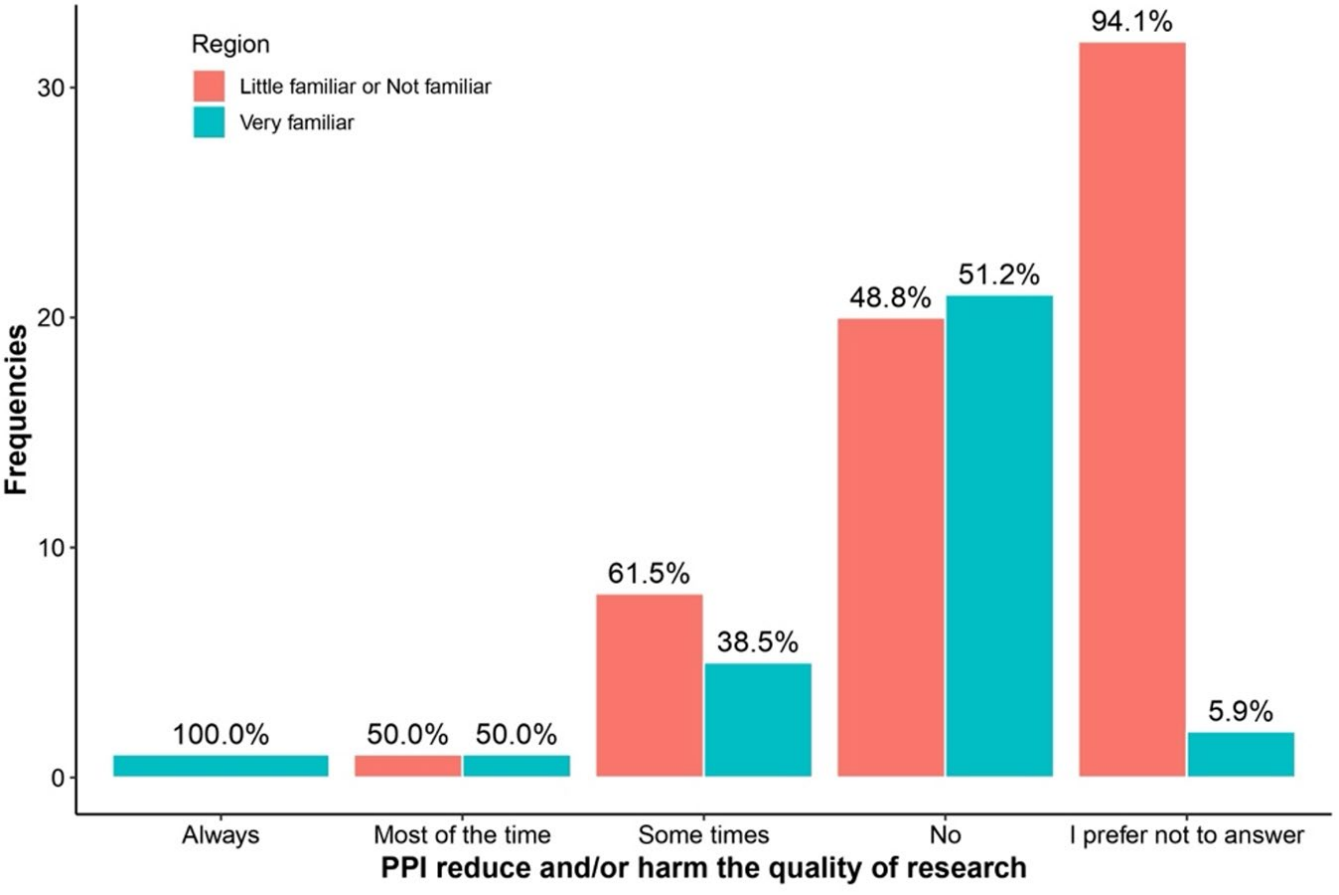
Knowledge of PPI and differences on the perception of PPI with respect to: Harm to research.

Table 3 highlights differences in responses between regions and levels of familiarity with PPI regarding the answer “I prefer not to answer.” Respondents more familiar with PPI were consistently less likely to select “I prefer not to answer” across all questions. This included questions about PPI availability (0% vs. 11.5%, *p* = 0.007), recruitment impact (13.3% vs. 60.7%, *p* < 0.001), research quality improvement (3.3% vs. 60.7%, *p* < 0.001), and potential harm to research (6.7% vs. 52.5%, *p* < 0.001). See Figs. 1, 2, 3, 4, 5, 6, 7 and 8.

The Figs. 1, 2, 3 and 4 compare responses from Europe/USA/Canada (Red) and Latin-America/Caribbean (blue). Europe/USA/Canada participants more often rated PPI as “Always” improving research “Quality” (75%) and “Inclusion” (90%), while Latin-America/Caribbean showed more distributed responses for Quality and more frequent “Sometimes” for “Inclusion” (72.7%). Recruitment was rated “Very good” in Europe/USA/Canada (72.7%) but “Not good” in Latin-America/Caribbean (93.3%). Both regions largely agreed that PPI does not harm research (80.5% AND 76.9%, respectively).

The bar charts in Figs. 5, 6, 7, 8 compare perceptions between “Very familiar (Blue) and “Little/Not familiar” (Red) groups. “Very familiar” respondents more often selected “Always” for Quality (87.5%) and Inclusion (70%), and “Very good” for recruitment (81.8%). “Little/Not familiar” respondents frequently chose “I prefer not to answer” (eg., 97.4% for Quality) and “Never” for Inclusion (96%). Both groups largely agreed that PPI does not harm research, although “Very familiar” respondents showed stronger agreement (61.5%).

**Fig. 9:**
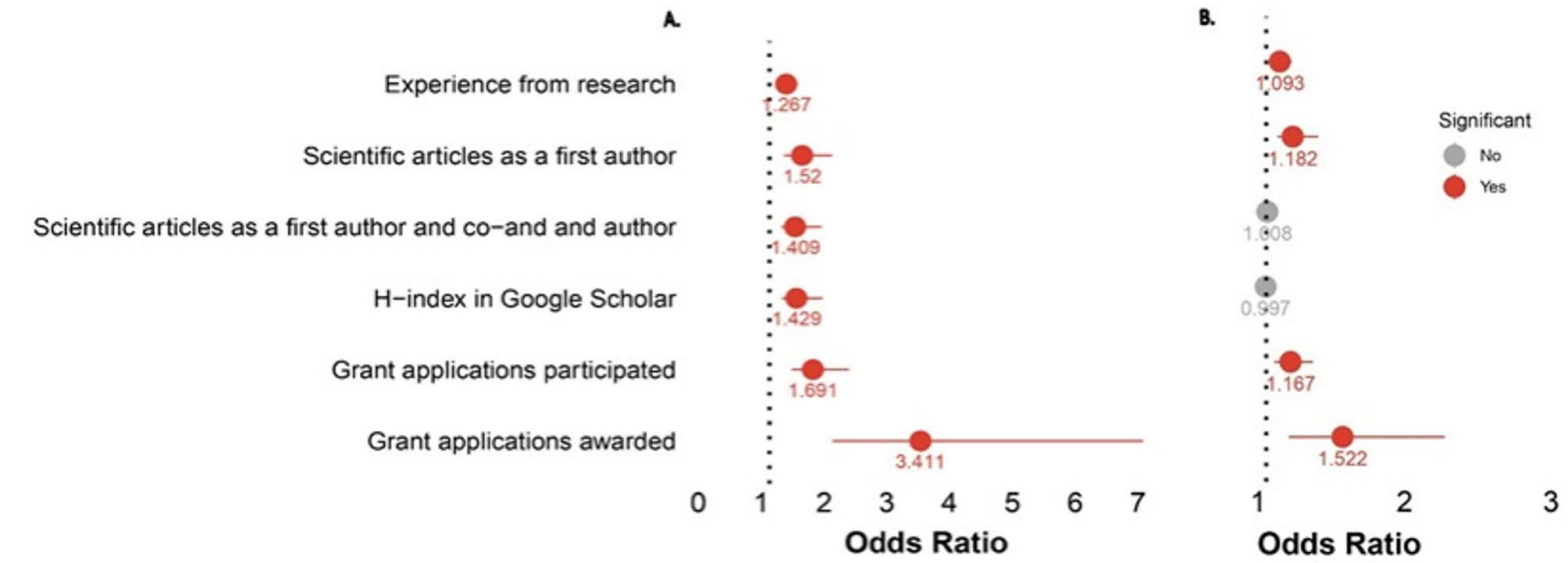
Logistic regression model (**A**) Latin-America/Caribbean vs. Europe/US/Canada (**B**) Familiarity.

The logistic regression model revealed that researchers from “Europe/USA/Canada” reported significantly more research experience (OR: 1.267, 95% CI: 1.138–1.448, *p* < 0.001), more first-author publications in the past five years (OR: 1.520, 95% CI: 1.228–1.995, *p* = 0.001), and a more significant total number of scientific articles (OR: 1.409, 95% CI: 1.185–1.828, *p* = 0.002). Additionally, they had submitted more grant applications in the past 36 months (OR: 1.691, 95% CI: 1.354–2.266, *p* < 0.001) and received more awarded grants (OR: 3.411, 95% CI: 2.000–6.954, *p* < 0.001). However, perceptions of PPI did not show significant results in this model (Fig. 9, and Supplementary Table S3).

The figure shows logistic regression models comparing (A) regions and (B) levels of familiarity with PPI. Six variables are analyzed: Research experience, Publications (first author/Co-author), H-index and Grant applications (participated and awarded). In (A), all variables are significant (Red), with Grant awards having the highest OR (3.411). In (B), Grant awards are significant (OR = 1.522), while H-index is non-significant (Gray, OR = 0.997).

In terms of PPI familiarity, those more familiar with PPI had more years of research experience (OR: 1.093, 95% CI: 1.024–1.176, *p* = 0.011) and more first-author publications in the past five years (OR: 1.182, 95% CI: 1.077–1.357, *p* = 0.004). They also participated in more grant applications over the last 36 months (OR: 1.167, 95% CI: 1.054–1.320, *p* = 0.006) and secured more successful grants (OR: 1.522, 95% CI: 1.155–2.219, *p* = 0.013). See Supplementary Table S4.

Familiarity with PPI was also strongly associated with having access to PPI groups (OR: 8.605, 95% CI: 2.910–30.012, *p* = 0.001) and using PPI to improve recruitment (OR: 99.145, 95% CI: 21.547–821.138, *p* < 0.001). No significant differences were found for other variables.

## Discussion

Recent literature corroborates the regional disparities and hesitancy seen in this study regarding PPI in dementia research. Persistent barriers include unfamiliarity with PPI concepts, lack of dementia-appropriate frameworks, and insufficient engagement of patients and caregivers—especially in under-resourced settings. Systematic reviews suggest PPI increases perceived study relevance and recruitment, but the PPI studies highlight the structural and cultural difficulties in implementing meaningful involvement and reporting outcomes clearly. Newer studies suggest that involving people living with dementia in co-designing research materials and decision-making can reduce misconceptions and stigma, improving autonomy and trust in research processes^20,36–39^.

A notable finding of this study is the high rate of non-responses or caution regarding questions related to PPI, particularly among respondents from “Latin America and the Caribbean.” These respondents were significantly more likely to select “I prefer not to answer” when asked about PPI’s impact on recruitment (63.3%) and research quality (60.0%). This could indicate uncertainty or discomfort with the concept of PPI in regions where it is less commonly implemented, or a lack of clear guidance on how to effectively integrate PPI into the research process. The same trend was observed among researchers less familiar with PPI, who were also more likely to opt for “I prefer not to answer”, coincidental with the 86.7% of the respondents from Latin America and the Caribbean which stated that they were little familiar or not familiar with PPI in research. This suggests that unfamiliarity with PPI could contribute to reluctance in assessing its effectiveness and highlighting the need for greater awareness and practical frameworks for integrating PPI into research workflows. Unfamiliarity with PPI can negatively affect the quality of research, hinder its effective implementation, and reduce the chances of securing international grants, where PPI is increasingly a requirement.

Additionally, we reported that researchers more familiar with PPI tend to have more research experience, first-author publications, and greater success in securing grants. This could suggest that experienced researchers are more familiar with the requirements of funding bodies, which increasingly mandate PPI in proposals. However, it is also possible that researchers who recognize the value of PPI are better equipped to produce impactful research and secure the funding necessary to support these activities.

Familiarity with PPI was strongly associated with accessing PPI groups and using PPI to improve recruitment. The striking odds ratio for PPI’s impact on recruitment suggested that researchers using PPI view it as a powerful tool for enhancing study participation. This aligns with existing literature suggesting that PPI could improve recruitment by making studies more relevant and accessible to the public, thus increasing the quality and applicability of research^11^. Despite the high association between familiarity with and use of PPI, no significant differences were found in perceptions of PPI’s impact on research quality, indicating the need for further investigation into how PPI influences research outcomes.

Additionally, significant regional differences in research experience, academic output, and engagement with PPI were observed, highlighting varied perceptions and applications of PPI across contexts. Researchers from “Europe/USA/Canada” had significantly more years of research experience, first-author publications, and greater grant success compared to their counterparts in “Latin America and the Caribbean.” These differences likely reflect disparities in research infrastructure, funding opportunities, and resources available in higher-income regions. The higher rate of awarded grants and applications in these regions underscores the competitive advantage that researchers in Europe and North America have in securing funding. It also highlights the potential challenges faced by researchers in under-resourced regions like Latin America in adopting PPI and other innovative research practices.

Another area of concern is the practical implementation of PPI in research. PPI implementation, execution and outcome metrics have not been established. Potential measurable effects may be developed in the future taking into consideration the societal effects of involving patients and the public in medical research^26^.

While 49.5% of respondents reported that their medical research institutions had groups available for PPI, a significant proportion (42.9%) had not yet utilized PPI to improve recruitment, and 45.1% expressed uncertainty about its effectiveness for this purpose. The study did not detail the composition of these PPI groups, leaving open the question of whether a broader selection of PPI participants (including patients, healthy individuals, healthcare workers, and researchers from different disciplines) might increase PPI’s perceived desirability and utility. This disconnect between the theoretical benefits of PPI and its practical application suggests that, while PPI is recognized as valuable, its implementation in research remains inconsistent.

The benefits of PPI have been studied and documented hereunder in dementia research subfields. Case studies from the Dementia and Neurodegenerative Diseases Research Network (DeNDRoN) illustrate how implementation of a structured group-based PPI program may correct issues in studies facing recruitment challenges or conceptual misunderstandings. For example, the researchers in the DOMINO-AD study were able to resolve recruitment issues, while the MUSTARDD-PD study benefitted from PPI’s insights into language use and risk assessment^40^. Similarly, the ROADMAN project collaborated with the European Dementia Working Group (EWGPW) to refine the survey design, making it more accessible and relevant to people with dementia^41^. Other studies, like Caregiving HOPE, engaged minority communities to enhance recruitment and representation, while the PREVENT study successfully used PPI to improve recruitment through patient ambassadors^42,43^. These examples underscore PPI’s potential efficacy to enhance study design, recruitment, and patient-centeredness.

PPI also influences research guidelines and outputs by ensuring the inclusion of patient perspectives in dementia research^44^ by involving patients in guideline development to achieve relevant recommendations for dementia diagnostics^45^, and by involving people with dementia as co-researchers to enhance data analysis validity. The SHARED study used PPI to co-create care planning recommendations for memory loss patients and their caregivers^46^. These studies highlight how PPI enhances the applicability and credibility of research findings by integrating the lived experiences of those affected by dementia.

The benefits of PPI extend beyond research design and recruitment to the participants themselves. In the PREVENT study, caregivers reported increased knowledge about dementia, which helped them in their caregiving roles^42^. Similarly^47^, found that PPI participation gave patients and caregivers a sense of purpose and satisfaction, fostering community ties. These outcomes demonstrate the potential reciprocal benefits of PPI—improving both research quality and the experiences of all involved. However, to capture and evaluate these benefits more effectively, additional research is needed. Such efforts should focus on clarifying the roles of user participants, establishing clear guidelines for implementing PPI, developing appropriate metrics and standards, defining funder requirements, and determining how best to allocate research time and funding to PPI activities^11,12,43^.

The high proportion of “I prefer not to answer” responses points to a need for clearer guidance on PPI, including who it impacts, what it impacts, how it works, and why it matters. Addressing researchers’ uncertainties and reservations about PPI will be crucial for its successful implementation and for realizing its full potential in aging and dementia research.

Several structured health care activities aim to enable researchers to implement PPI. Some medical and graduate programs include modules on public involvement and research co-production. For practicing researchers, national and international bodies provide short courses, workshops, online learning, and practical toolkits that cover planning, role definition, compensation, governance, and evaluation Evaluations of these offerings generally suggests gains in knowledge, confidence, and intentions to use PPI, but evidence for sustained practice change, integration into study governance, and downstream impact on recruitment, retention, or outcomes is heterogeneous and often based on self-report. In dementia research specifically, adaptations emphasize involvement of people with cognitive impairment and care partners; however, formal evaluations of dementia-focused training remain scarce. Taken together, the training landscape is growing but uneven, underscoring the need for standardized competency frameworks, condition-specific guidance, and longitudinal evaluation^27^. The results of this study underscore the need for targeted education and facilitate a meaningful dialogue between a wider range of researchers and funders on PPI, particularly regarding how it can be effectively integrated into research processes to enhance recruitment, implement objective quality metrics for PPI, and improve public engagement in scientific research^26^. Moreover, future studies should examine how PPI familiarity evolves, how to engage the public and public decision-makers to a greater extent^26^, how to standardize and measure training interventions, and explore how factors such as gender and academic discipline, culture and socioeconomics influence attitudes towards PPI.

## Limitations and strengths

This study has several limitations that may affect the generalizability and applicability of the findings. Firstly, the response rate of 23.2% could introduce response bias. The survey was anonymous, and the population composition is not known. Therefore, the sample used in this survey may therefore not be representative of the population. This is a limitation to the generalizability of the study, e.g. those who chose to participate may have had stronger opinions and/or different perceptions on PPI compared to those who did not respond.

However, this response rate is relatively good for online studies, particularly when involving specialized populations like researchers^48^. Additionally, the cross-sectional design limits the ability to draw causal conclusions about the relationship between PPI familiarity and research outcomes. We acknowledge that longitudinal studies could provide better insights into how familiarity with PPI evolves over time and impacts research outcomes. The reliance on self-reported data introduces potential recall bias or social desirability bias, particularly for sensitive questions related to PPI implementation. Additionally, variables with very large ORs observed in the results can be attributed to the small number of cases in specific categories. Another limitation is this study’s dichotomized answer options and exclusive focus on quantitative data limits the depth of insights into researchers’ attitudes toward PPI, underscoring the need for qualitative follow-up studies to capture a more detailed and individualized perspective and identify barriers to PPI. In addition, further investigations that can examine how factors like gender, socioeconomic status, and research discipline influence attitudes toward PPI. Furthermore, it was beyond the time and recourses available to this study to include global regions outside Americas and Europe. The regional focus of this study on Europe, Latin America, and North America, and the grouping of Europe/USA/Canada and Latin America, restricts the applicability of the findings to other global regions where PPI practices and research infrastructures may differ significantly. Future research should prioritize underrepresented regions such as Asia and Africa to provide a more global perspective.

On the other hand, this study offers several strengths. To our knowledge, it is the first paper to address this critical and timely topic, exploring an area of growing concern. The multinational approach, which includes participants from Europe, Latin America, Canada, and the United States, allows for meaningful cross-regional comparisons, shedding light on disparities in PPI practices between high-income and under-resourced areas. The comprehensive survey design ensured a thorough understanding of participants’ views, and the multi-step validation process—including expert reviews and feedback from focus groups—enhanced the reliability of the questionnaire. Ethical considerations, such as participant anonymity and the conduct of a pilot test, further strengthen the study’s credibility.

### Future research

In the past PPI has been studied from an institutional level and with respect to the researcher-patient/participant relationship. This study has a novel aim by focusing on the researchers’ own perception of PPI across different counties in Europe and Americas. The adoption of PPI in research varies substantially across global regions, between individual countries within these regions. While Europe and North America have developed institutional frameworks and funding strategies that encourage and support PPI. Countries on other regions such as Asia, Africa, the Middle East, and Oceania may face structural and cultural barriers. In these areas, limited policy mandates, resource constraints, hierarchies within healthcare and research environments, and lower levels of awareness about the benefits of participatory research may contribute to the slower uptake of PPI. Additionally, reporting on PPI activities, where they do occur, often lacks rigor and depth, further hindering knowledge transfer and capacity-building for effective PPI models in these regions. In Latin America, PPI remains limited due to the absence of funding requirements or policy incentives, entrenched researcher-centered traditions that privilege biomedical expertise, and structural constraints such as scarce resources and fragmented health systems^20,27,36,49^. Future studies may consider repeating the study in other geographic areas and different medical fields to explore the nuanced implementation of patient and public involvement in practice, hereunder exploring the impact of different structural and resource barriers/enablers, cultural and institutional factors, diversity and strategic considerations. Other studies could seek to understand the high number of respondents preferring not to answer PPI questions.

## Conclusion

Earlier studies have undertaken to explore theories, enablers and barriers to PPI. Despite state sponsored PPI programs for more than 50 years^50^, implementation of PPI is complex and continues to be fragmented, and the results from PPI remains unclear^27,51,52^. The study’s findings hold important implications for research funders and policymakers. As PPI increasingly becomes a requirement set by funding agencies, it is crucial to address regional disparities in both understanding and applying PPI in a structured, evidence-based manner. Researchers in high-income countries may have more time, resources, and experience to implement PPI effectively, whereas those in lower-income regions may need additional, targeted support and guidance to fully realize its benefits. Policymakers and research institutions should focus on providing training and resources to encourage PPI adoption, particularly where it is less well-known.

## Supplementary Information

Below is the link to the electronic supplementary material.

Supplementary Material 1

## Data Availability

Data used in this study will be available upon request to the corresponding author.

## Abbreviations

PPI: Patient and Public Involvement
CPG: Clinical practice guidelines
RRI: Responsible research and innovation
EU: European Union
GDPR: General data protection regulation
SESAM: Centre for age-related medicine (Stavanger University Hospital)
DeNDRoN: Dementias & neurodegenerative diseases research network
EWGPWD: European Working Group of People with Dementia
PREVENT: Prevent Dementia Programme
SHARED: Study of Health and Research Engagement in Dementia
OR: Odds ratio
CI: Confidence interval

## References

[1] Carrier, M. & Gartzlaff, M. Responsible research and innovation: hopes and fears in the scientific community in Europe. *J. Responsible Innov.* **7**(2), 149–169 (2020).

[2] Owen, R. et al. A framework for responsible innovation. *Responsible Innov. Manag. Responsible Emerg. Sci. Innov. Soc.* 27–50 (2013).

[3] Von Schomberg, R. A vision of responsible research and innovation. In Responsible Innovation (eds Owen, R. et al.) 51–74 (Wiley, 2013).

[4] European Commission. *Dissemination and Exploitation of Research Results* http://research-and-innovation.ec.europa.eu/strategy/dissemination-and-exploitation-research-results_en (European Commission, 2024).

[5] European Research Executive Agency. Communication about your EU-funded project http://rea.ec.europa.eu/communicating-about-your-eu-funded-project_en, https://rea.ec.europa.eu/communicating-about-your-eu-funded-project_en#acknowledge-eu-funding (European Commission, 2024).

[6] Aries, A. M., Bailey, P. & Hunter, S. M. The mutual benefits of patient and public involvement in research: an example from a feasibility study (MoTaStim-Foot). *Res. Involv. Engagem.* **7**, 1–14 (2021). 34863297 10.1186/s40900-021-00330-w PMC8645133

[7] Bryant, E. A., Scott, A. M., Greenwood, H. & Thomas, R. Patient and public involvement in the development of clinical practice guidelines: a scoping review. *BMJ open.* **12**(9), e055428 (2022). 36171042 10.1136/bmjopen-2021-055428 PMC9528587

[8] Armstrong, M. J., Rueda, J. D., Gronseth, G. S. & Mullins, C. D. Framework for enhancing clinical practice guidelines through continuous patient engagement. *Health Expect.* **20**(1), 3–10 (2017). 27115476 10.1111/hex.12467 PMC5217879

[9] Kylén, M., Slaug, B., Jonsson, O., Iwarsson, S. & Schmidt, S. M. User involvement in ageing and health research: a survey of researchers’ and older adults’ perspectives. *Health Res. Policy Syst.* **20**(1), 93 (2022). 36050697 10.1186/s12961-022-00894-3 PMC9438331

[10] Malterud, K. & Elvbakken, K. T. Patients participating as co-researchers in health research: a systematic review of outcomes and experiences. *Scand. J. Public Health*. **48**(6), 617–628 (2020). 31319762 10.1177/1403494819863514

[11] Aas, S. N. et al. Patient and public involvement in health research in norway: a survey among researchers and patient organisations. *Res. Involv. Engagem.* **9**(1), 48 (2023). 37422661 10.1186/s40900-023-00458-x PMC10329785

[12] Blackburn, S. et al. The extent, quality and impact of patient and public involvement in primary care research: a mixed methods study. *Res. Involv. Engagem.* **4**, 1–18 (2018). 29850029 10.1186/s40900-018-0100-8 PMC5966874

[13] Rogers, P. J. Using programme theory to evaluate complicated and complex aspects of interventions. *Evaluation* **14**(1), 29–48 (2008).

[14] Hinrichs-Krapels, S. & Grant, J. Exploring the effectiveness, efficiency and equity (3e’s) of research and research impact assessment. *Palgrave Commun.* **2**(1), 1–9 (2016).

[15] Viergever, R. F. The mismatch between the health research and development (R&D) that is needed and the R&D that is undertaken: an overview of the problem, the causes, and solutions. *Global Health Action*. **6**(1), 22450 (2013). 24119660 10.3402/gha.v6i0.22450 PMC3796018

[16] Oliver, K. & Cairney, P. The dos and don’ts of influencing policy: a systematic review of advice to academics. *Palgrave Commun.* **5**(1), 1–11 (2019).

[17] Otten, J. J., Dodson, E. A., Fleischhacker, S., Siddiqi, S. & Quinn, E. L. Peer reviewed: getting research to the policy table: a qualitative study with public health researchers on engaging with policy makers. *Prev. Chronic Dis.* **12**(2015). 10.5888/pcd12.140546 PMC4416480 25927604

[18] Boylan, A. M., Locock, L., Thomson, R. & Staniszewska, S. About sixty per cent I want to do it: health researchers’ attitudes to, and experiences of, patient and public involvement (PPI)—A qualitative interview study. *Health Expect.* **22**(4), 721–730 (2019). 30927334 10.1111/hex.12883 PMC6737750

[19] Foster, A. et al. Evaluating a grant development public involvement funding scheme: a qualitative document analysis. *Res. Involv. Engagem.* **10**(1), 57 (2024). 38858792 10.1186/s40900-024-00588-w PMC11163746

[20] Lang, I. et al. How common is patient and public involvement (PPI)? Cross-sectional analysis of frequency of PPI reporting in health research papers and associations with methods, funding sources and other factors. *BMJ open.* **12**(5), e063356 (2022). 35613748 10.1136/bmjopen-2022-063356 PMC9131100

[21] Agyei-Manu, E. et al. The benefits, challenges, and best practice for patient and public involvement in evidence synthesis: A systematic review and thematic synthesis. *Health Expect.* **26**(4), 1436–1452 (2023). 37260191 10.1111/hex.13787 PMC10349234

[22] Carroll, P. et al. The role of patient and public involvement (PPI) in pre-clinical spinal cord research: an interview study. *Plos One*. **19**(4), e0301626 (2024). 38683786 10.1371/journal.pone.0301626 PMC11057720

[23] Vinnicombe, S., Bianchim, M. S. & Noyes, J. A review of reviews exploring patient and public involvement in population health research and development of tools containing best practice guidance. *BMC Public. Health*. **23**(1), 1271 (2023). 37391764 10.1186/s12889-023-15937-9 PMC10311710

[24] Predictom.eu. How is Public Involvement organised in PREDICTOM? https://www.predictom.eu/public-involvement-user-impact-in-predictom (Predictom.eu, 2025).

[25] Helse Stavanger. WiseAge www.helse-stavanger.no/en/wiseage-en/ (Helse Stavanger, 2025).

[26] Fredriksson, M., Sampaio, F. & Moberg, L. The impact of patient and public involvement in healthcare services: A conceptual review spanning social sciences and health sciences. *SSM-Qualitative Res. Health*. **7**, 100517 (2025).

[27] Ocloo, J., Garfield, S., Franklin, B. D. & Dawson, S. Exploring the theory, barriers and enablers for patient and public involvement across health, social care and patient safety: a systematic review of reviews. *Health Res. Policy Syst.* **19**(1), 8 (2021). 33472647 10.1186/s12961-020-00644-3 PMC7816359

[28] World Health Organization. A blueprint for dementia research https://www.who.int/publications/i/item/9789240058248 (World Health Organizatio, 2022).

[29] Allegri, R. F. Dementia research in low-income and middle-income countries—a view from Latin America. *Nat. Rev. Neurol.* 1–7 (2025). 10.1038/s41582-025-01125-3 40730880

[30] Fusdahl, P. et al. Perspectives of old-age and dementia researchers on communication with policymakers and public research funding decision-makers: an international cross-sectional survey. *Front. Med.* **11** (2024). 10.3389/fmed.2024.1472479 PMC11695358 39760038

[31] The World Bank. World Bank Country and Lending Groups http://datahelpdesk.worldbank.org/knowledgebase/articles/906519-world-bank-country-and-lending-groups (The World Bank, 2025).

[32] University of Oslo. Short introduction to Nettskjema https://www.uio.no/english/services/it/adm-services/nettskjema/about-nettskjema.html (University of Oslo, 2023).

[33] Emmanuel, A. & Clow, S. E. A questionnaire for assessing breastfeeding intentions and practices in nigeria: validity, reliability and translation. *BMC Pregnancy Childbirth*. **17**(1), 1–7 (2017). 28592252 10.1186/s12884-017-1366-9 PMC5463374

[34] Lim, W. M. A typology of validity: content, face, convergent, discriminant, nomological and predictive validity. *J. Trade Sci.* **12**(3), 155–179 (2024).

[35] Haynes, W. Benjamini–hochberg method. *Encyclopedia Syst. Biol.* **78** (2013).

[36] Cook, N., Siddiqi, N., Twiddy, M. & Kenyon, R. Patient and public involvement in health research in low and middle-income countries: a systematic review. *BMJ open.* **9**(5), e026514 (2019). 31076471 10.1136/bmjopen-2018-026514 PMC6528003

[37] Walter, S. et al. Public and participant involvement as a pathway to inclusive dementia research. *Alzheimer’s Dement.* **21**(1), e14350 (2025). 39540563 10.1002/alz.14350 PMC11782197

[38] Yang, W. S. et al. Evaluation of the impact of patient and public involvement on doctoral students in palliative dementia care research. *Res. Involv. Engagem.* **11**(1), 73 (2025). 40611227 10.1186/s40900-025-00715-1 PMC12224461

[39] Zhou, C. et al. Barriers and facilitators to participation in electronic health interventions in older adults with cognitive impairment: an umbrella review. *BMC Geriatr.* **24**(1), 1037 (2024). 39725926 10.1186/s12877-024-05645-3 PMC11670401

[40] Iliffe, S., McGrath, T. & Mitchell, D. The impact of patient and public involvement in the work of the dementias & neurodegenerative diseases research network (DeNDRoN): case studies. *Health Expect.* **16**(4), 351–361 (2013). 21902772 10.1111/j.1369-7625.2011.00728.x PMC5060691

[41] Diaz, A. et al. Conducting public involvement in dementia research: the contribution of the European working group of people with dementia to the ROADMAP project. *Health Expect.* **24**(3), 757–765 (2021). 33822448 10.1111/hex.13246 PMC8235878

[42] Gregory, S. et al. Research participants as collaborators: background, experience and policies from the PREVENT dementia and EPAD programmes. *Dementia* **17**(8), 1045–1054 (2018). 30373458 10.1177/1471301218789307

[43] Parveen, S. et al. Involving minority ethnic communities and diverse experts by experience in dementia research: the caregiving HOPE study. *Dementia* **17**(8), 990–1000 (2018). 30373461 10.1177/1471301218789558

[44] Armstrong, M. J., Mullins, C. D., Gronseth, G. S. & Gagliardi, A. R. Impact of patient involvement on clinical practice guideline development: a parallel group study. *Implement. Sci.* **13**, 1–13 (2018). 29661195 10.1186/s13012-018-0745-6 PMC5902835

[45] Stevenson, M. & Taylor, B. J. Involving individuals with dementia as co-researchers in analysis of findings from a qualitative study. *Dementia* **18**(2), 701–712 (2019). 28133983 10.1177/1471301217690904

[46] Mockford, C. et al. A SHARED study-the benefits and costs of setting up a health research study involving lay co-researchers and how we overcame the challenges. *Res. Involv. Engagem.* **2**, 1–12 (2016). 29062509 10.1186/s40900-016-0021-3 PMC5611649

[47] Miah, J. et al. Impact of involving people with dementia and their care partners in research: a qualitative study. *BMJ open.* **10**(10), e039321 (2020). 33109666 10.1136/bmjopen-2020-039321 PMC7592301

[48] Wu, M-J., Zhao, K. & Fils-Aime, F. Response rates of online surveys in published research: A meta-analysis. *Computers Hum. Behav. Rep.* **7**, 100206 (2022).

[49] Troya, M. I., Bartlam, B. & Chew-Graham, C. A. Involving the public in health research in Latin America: making the case for mental health. *Revista Panam. De Salud Pública*. **42**, e45 (2018). 10.26633/RPSP.2018.45 PMC6386097 31093073

[50] Hogg, C. N. Patient and public involvement: what next for the NHS? *Health Expect.* **10**(2), 129–138 (2007). 17524006 10.1111/j.1369-7625.2006.00427.x PMC5060383

[51] Pierce, M. et al. Embedding formal and experiential public and patient involvement training in a structured phd programme: process and impact evaluation. *Res. Involv. Engagem.* **9**(1), 105 (2023). 37996882 10.1186/s40900-023-00516-4 PMC10668398

[52] Yu, R., Hanley, B., Denegri, S., Ahmed, J. & McNally, N. J. Evaluation of a patient and public involvement training programme for researchers at a large biomedical research centre in the UK. *BMJ open.* **11**(8), e047995 (2021). 34385250 10.1136/bmjopen-2020-047995 PMC8362711

