# Supplementary material for: An international cross-sectional study of dementia researchers’ own perspectives on patient and public involvement
Source: Sci Rep. 2025 Dec 29;16:3846. doi: 10.1038/s41598-025-33955-y (PMC12852740; doi:10.1038/s41598-025-33955-y)
Supplement: Supplementary file 1 — Supplementary Material 1 [file 41598_2025_33955_MOESM1_ESM.docx]

**Supplementary Material Table S1.** Model evaluating differences in the research characteristics and opinions according to region and knowledge about Patient and Public Involvement**.**

| **Variables** | **Overall** | **Europe/USA/Canada** | **Latin America and Caribbean** |  | **P value** |
| --- | --- | --- | --- | --- | --- |
|  | **(n = 91)** | **(n = 61)** | **(n = 30)** |  |  |
| **How many years of experience from research do you have?** |  |  |  |  |  |
| Mean (SD) | 15.1 (10.2) | 16.8 (10.4) | 11.6 (8.95) |  |  |
| Median [Q1, Q3] | 14.0 [8.00, 20.0] | 15.0 [10.0, 20.0] | 10.0 [4.00, 19.5] |  | 0.019 |
| [MIN, MAX] | [1.00, 50.0] | [1.00, 50.0] | [1.00, 30.0] |  |  |
| **How many scientific articles were you first author of the last 5 years?** |  |  |  |  |  |
| Mean (SD) | 12.5 (23.4) | 16.2 (27.4) | 5.07 (7.91) |  |  |
| Median [Q1, Q3] | 5.00 [2.00, 10.0] | 7.00 [3.00, 15.0] | 3.00 [0, 5.00] |  | 0.001 |
| [MIN, MAX] | [0, 170] | [0, 170] | [0, 40.0] |  |  |
| **How many scientific articles have you written as first author and co-author in your whole career?** |  |  |  |  |  |
| Mean (SD) | 90.6 (206) | 120 (244) | 30.4 (47.2) |  |  |
| Median [Q1, Q3] | 31.0 [10.0, 80.0] | 42.0 [17.0, 110] | 10.0 [3.00, 45.3] |  | < 0.001 |
| [MIN, MAX] | [0, 1500] | [1.00, 1500] | [0, 180] |  |  |
| **What is your H-index in Google Scholar last 5 years (since 2018)?** |  |  |  |  |  |
| Mean (SD) | 25.2 (31.1) | 33.4 (34.4) | 8.73 (11.8) |  |  |
| Median [Q1, Q3] | 16.0 [2.50, 33.5] | 26.0 [10.0, 46.0] | 2.00 [0, 16.0] |  | < 0.001 |
| [MIN, MAX] | [0, 146] | [0, 146] | [0, 36.0] |  |  |
| **How many grant applications have you participated in in the last 36 months months?** |  |  |  |  |  |
| Mean (SD) | 7.95 (10.3) | 10.8 (11.5) | 2.17 (2.34) |  |  |
| Median [Q1, Q3] | 5.00 [2.00, 10.0] | 8.00 [4.00, 11.0] | 1.50 [0, 3.75] |  | < 0.001 |
| [MIN, MAX] | [0, 53.0] | [0, 53.0] | [0, 8.00] |  |  |
| **How many of the grant applications you participated in were awarded funding in the last 36 months?** |  |  |  |  |  |
| Mean (SD) | 3.88 (5.79) | 5.21 (6.62) | 1.17 (1.39) |  |  |
| Median [Q1, Q3] | 2.00 [1.00, 4.00] | 3.00 [2.00, 5.00] | 1.00 [0, 1.00] |  | < 0.001 |
| [MIN, MAX] | [0, 40.0] | [0, 40.0] | [0, 5.00] |  |  |
| **Are you familiar with Patient and Public Involvement (PPI) in research?** |  |  |  |  |  |
| Little familiar or Not familiar | 61 (67.0%) | 35 (57.4%) | 26 (86.7%) |  | 0.011 |
| Very familiar | 30 (33.0%) | 26 (42.6%) | 4 (13.3%) |  |  |
| **Does your research institution have groups of people that are available for PPI in your research?** |  |  |  |  |  |
| Yes | 45 (49.5%) | 33 (54.1%) | 12 (40.0%) |  | 0.438 |
| No | 39 (42.9%) | 24 (39.3%) | 15 (50.0%) |  |  |
| I prefer not to answer | 7 (7.7%) | 4 (6.6%) | 3 (10.0%) |  |  |
| **Have you used PPI to improve recruitment of participants in research and clinical trials?** |  |  |  |  |  |
| Yes | 25 (27.5%) | 21 (34.4%) | 4 (13.3%) |  | 0.062 |
| No | 66 (72.5%) | 40 (65.6%) | 26 (86.7%) |  |  |
| **How do you think PPI works in the recruitment of participants for research and clinical trials?** |  |  |  |  |  |
| 1. Very good | 11 (12.1%) | 8 (13.1%) | 3 (10.0%) |  | 0.040 |
| 2. Good | 24 (26.4%) | 17 (27.9%) | 7 (23.3%) |  |  |
| 3. Not good | 15 (16.5%) | 14 (23.0%) | 1 (3.3%) |  |  |
| 4. I prefer not to answer | 41 (45.1%) | 22 (36.1%) | 19 (63.3%) |  |  |
| **Does PPI improve the quality of your research?** |  |  |  |  |  |
| 1. Always | 8 (8.8%) | 6 (9.8%) | 2 (6.7%) |  | 0.039 |
| 2. Most of the time | 16 (17.6%) | 10 (16.4%) | 6 (20.0%) |  |  |
| 3. Some times | 19 (20.9%) | 15 (24.6%) | 4 (13.3%) |  |  |
| 4. No | 10 (11.0%) | 10 (16.4%) | 0 (0%) |  |  |
| 5. I prefer not to answer | 38 (41.8%) | 20 (32.8%) | 18 (60.0%) |  |  |
| **Do PPI reduce and/or harm the quality of your research?** |  |  |  |  |  |
| 1. Always | 1 (1.1%) | 1 (1.6%) | 0 (0%) |  | 0.011 |
| 2. Most of the time | 2 (2.2%) | 0 (0%) | 2 (6.7%) |  |  |
| 3. Some times | 13 (14.3%) | 10 (16.4%) | 3 (10.0%) |  |  |
| 4. No | 41 (45.1%) | 33 (54.1%) | 8 (26.7%) |  |  |
| 5. I prefer not to answer | 34 (37.4%) | 17 (27.9%) | 17 (56.7%) |  |  |
| **How often do you include PPI in your more recent research projects and/or articles?** |  |  |  |  |  |
| 1. Always | 10 (11.0%) | 9 (14.8%) | 1 (3.3%) |  | 0.019 |
| 2. Most of the time | 14 (15.4%) | 10 (16.4%) | 4 (13.3%) |  |  |
| 3. Some times | 22 (24.2%) | 16 (26.2%) | 6 (20.0%) |  |  |
| 4. Never | 18 (19.8%) | 15 (24.6%) | 3 (10.0%) |  |  |
| 5. Not relevant | 2 (2.2%) | 1 (1.6%) | 1 (3.3%) |  |  |
| 6. I prefer not to answer | 25 (27.5%) | 10 (16.4%) | 15 (50.0%) |  |  |

**Supplementary Material Table S2.** Bivariate analysis evaluating differences in the research characteristics and opinions according to familiarity and knowledge about Patient and Public Involvement**.**

| **Variables** | **Overall** | **Little familiar or Not familiar** | **Very familiar** | **P value** |
| --- | --- | --- | --- | --- |
|  | **(n = 91)** | **(n = 61)** | **(n = 30)** |  |
| **How many years of experience from research do you have?** |  |  |  |  |
| Mean (SD) | 15.1 (10.2) | 13.9 (11.2) | 17.5 (7.45) |  |
| Median [Q1, Q3] | 14.0 [8.00, 20.0] | 12.0 [5.00, 20.0] | 16.0 [13.3, 20.0] | 0.014 |
| [MIN, MAX] | [1.00, 50.0] | [1.00, 50.0] | [6.00, 40.0] |  |
| **How many scientific articles were you first author of the last 5 years?** |  |  |  |  |
| Mean (SD) | 12.5 (23.4) | 7.61 (14.6) | 22.5 (33.2) |  |
| Median [Q1, Q3] | 5.00 [2.00, 10.0] | 4.00 [2.00, 8.00] | 10.0 [5.00, 29.5] | 0.001 |
| [MIN, MAX] | [0, 170] | [0, 100] | [0, 170] |  |
| **How many scientific articles have you written as first author and co-author in your whole career?** |  |  |  |  |
| Mean (SD) | 90.6 (206) | 81.9 (230) | 108 (147) |  |
| Median [Q1, Q3] | 31.0 [10.0, 80.0] | 20.0 [5.00, 52.0] | 51.5 [29.3, 139] | 0.001 |
| [MIN, MAX] | [0, 1500] | [0, 1500] | [6.00, 750] |  |
| **What is your H-index in Google Scholar last 5 years (since 2018)?** |  |  |  |  |
| Mean (SD) | 25.2 (31.1) | 23.1 (34.9) | 29.6 (21.6) |  |
| Median [Q1, Q3] | 16.0 [2.50, 33.5] | 9.00 [2.00, 30.0] | 27.0 [11.3, 40.8] | 0.008 |
| [MIN, MAX] | [0, 146] | [0, 146] | [0, 80.0] |  |
| **How many grant applications have you participated in in the last 36 months months?** |  |  |  |  |
| Mean (SD) | 7.95 (10.3) | 6.49 (10.2) | 10.9 (10.1) |  |
| Median [Q1, Q3] | 5.00 [2.00, 10.0] | 3.00 [1.00, 8.00] | 8.00 [5.00, 14.0] | < 0.001 |
| [MIN, MAX] | [0, 53.0] | [0, 52.0] | [1.00, 53.0] |  |
| **How many of the grant applications you participated in were awarded funding in the last 36 months?** |  |  |  |  |
| Mean (SD) | 3.88 (5.79) | 3.30 (6.01) | 5.07 (5.20) |  |
| Median [Q1, Q3] | 2.00 [1.00, 4.00] | 1.00 [0, 4.00] | 4.00 [2.00, 5.75] | 0.002 |
| [MIN, MAX] | [0, 40.0] | [0, 40.0] | [0, 21.0] |  |
| **Does your research institution have groups of people that are available for PPI in your research?** |  |  |  |  |
| I prefer not to answer | 7 (7.7%) | 7 (11.5%) | 0 (0%) | < 0.001 |
| No | 39 (42.9%) | 34 (55.7%) | 5 (16.7%) |  |
| Yes | 45 (49.5%) | 20 (32.8%) | 25 (83.3%) |  |
| **Have you used PPI to improve recruitment of participants in research and clinical trials?** |  |  |  |  |
| No | 66 (72.5%) | 59 (96.7%) | 7 (23.3%) | < 0.001 |
| Yes | 25 (27.5%) | 2 (3.3%) | 23 (76.7%) |  |
| **How do you think PPI works in the recruitment of participants for research and clinical trials?** |  |  |  |  |
| 1. Very good | 11 (12.1%) | 2 (3.3%) | 9 (30.0%) | < 0.001 |
| 2. Good | 24 (26.4%) | 12 (19.7%) | 12 (40.0%) |  |
| 3. Not good | 15 (16.5%) | 10 (16.4%) | 5 (16.7%) |  |
| 4. I prefer not to answer | 41 (45.1%) | 37 (60.7%) | 4 (13.3%) |  |
| **Does PPI improve the quality of your research?** |  |  |  |  |
| 1. Always | 8 (8.8%) | 1 (1.6%) | 7 (23.3%) | 0.001 |
| 2. Most of the time | 16 (17.6%) | 6 (9.8%) | 10 (33.3%) |  |
| 3. Some times | 19 (20.9%) | 10 (16.4%) | 9 (30.0%) |  |
| 4. No | 10 (11.0%) | 7 (11.5%) | 3 (10.0%) |  |
| 5. I prefer not to answer | 38 (41.8%) | 37 (60.7%) | 1 (3.3%) |  |
| **Do PPI reduce and/or harm the quality of your research?** |  |  |  |  |
| 1. Always | 1 (1.1%) | 0 (0%) | 1 (3.3%) | 0.001 |
| 2. Most of the time | 2 (2.2%) | 1 (1.6%) | 1 (3.3%) |  |
| 3. Some times | 13 (14.3%) | 8 (13.1%) | 5 (16.7%) |  |
| 4. No | 41 (45.1%) | 20 (32.8%) | 21 (70.0%) |  |
| 5. I prefer not to answer | 34 (37.4%) | 32 (52.5%) | 2 (6.7%) |  |
| **How often do you include PPI in your more recent research projects and/or articles?** |  |  |  |  |
| 1. Always | 10 (11.0%) | 3 (4.9%) | 7 (23.3%) | < 0.001 |
| 2. Most of the time | 14 (15.4%) | 1 (1.6%) | 13 (43.3%) |  |
| 3. Some times | 22 (24.2%) | 13 (21.3%) | 9 (30.0%) |  |
| 4. Never | 18 (19.8%) | 18 (29.5%) | 0 (0%) |  |
| 5. Not relevant | 2 (2.2%) | 2 (3.3%) | 0 (0%) |  |
| 6. I prefer not to answer | 25 (27.5%) | 24 (39.3%) | 1 (3.3%) |  |

**Supplementary Material Table S3.** Logistic regression Patient and Public Involvement characteristics with region.

| **Exposure variable** | **OR** | **IC 95%** | | **p value** | **Adjusted p value** |
| --- | --- | --- | --- | --- | --- |
|  |  | **Lower** | **upper** |  |  |
| How many years of experience from research do you have? | 1.267 | 1.138 | 1.448 | < 0.001 | < 0.001 |
| How many scientific articles were you first author of the last 5 years? | 1.520 | 1.228 | 1.995 | 0.001 | 0.001 |
| How many scientific articles have you written as first author and co-author in your whole career? | 1.409 | 1.185 | 1.828 | 0.002 | 0.002 |
| What is your H-index in Google Scholar last 5 years (since 2018)? | 1.429 | 1.200 | 1.848 | 0.001 | 0.002 |
| How many grant applications have you participated in in the last 36 months months? | 1.691 | 1.354 | 2.266 | < 0.001 | < 0.001 |
| How many of the grant applications you participated in were awarded funding in the last 36 months? | 3.411 | 2.000 | 6.954 | < 0.001 | < 0.001 |
| Does your research institution have groups of people that are available for PPI in your research? | 1.719 | 0.848 | 3.117 | 0.250 | 0.521 |
| Have you used PPI to improve recruitment of participants in research and clinical trials? | 3.413 | 1.141 | 12.706 | 0.041 | 0.205 |
| How do you think PPI works in the recruitment of participants for research and clinical trials? | 0.179 | 0.009 | 1.082 | 0.118 | 0.463 |
| Does PPI improve the quality of your research? | - | - | - | - | - |
| Do PPI reduce and/or harm the quality of your research? | 0.533 | 0.145 | 2.074 | 0.347 | 0.694 |
| How often do you include PPI in your more recent research projects and/or articles? | 0.795 | 0.197 | 2.739 | 0.728 | 0.728 |

** Were excluded the responses "I prefer not to answer" in all models

Latin-America was taken as reference variable

'- Convergency problems related to the lack of observations in the category “Little familiar or not familiar” in the dependent variable

**Supplementary Material Table S4.** Logistic regression Patient and Public Involvement characteristics with Knowledge about Patient and Public Involvement.

| **Exposure variable** | **OR** | **IC 95%** | | **p value** | **Adjusted p value** |
| --- | --- | --- | --- | --- | --- |
|  |  | **Lower** | **upper** |  |  |
| How many years of experience from research do you have? | 1.093 | 1.024 | 1.176 | 0.011 | 0.032 |
| How many scientific articles were you first author of the last 5 years? | 1.182 | 1.077 | 1.357 | 0.004 | 0.020 |
| How many scientific articles have you written as first author and co-author in your whole career? | 1.008 | 1.001 | 1.017 | 0.084 | 0.168 |
| What is your H-index in Google Scholar last 5 years (since 2018)? | 0.997 | 0.982 | 1.012 | 0.742 | 0.742 |
| How many grant applications have you participated in in the last 36 months months? | 1.167 | 1.054 | 1.320 | 0.006 | 0.025 |
| How many of the grant applications you participated in were awarded funding in the last 36 months? | 1.522 | 1.155 | 2.219 | 0.013 | 0.039 |
| Does your research institution have groups of people that are available for PPI in your research? | 8.605 | 2.910 | 30.012 | < 0.001 | 0.001 |
| Have you used PPI to improve recruitment of participants in research and clinical trials? | 99.145 | 21.547 | 821.138 | < 0.001 | < 0.001 |
| How do you think PPI works in the recruitment of participants for research and clinical trials? | 4.088 | 1.108 | 17.031 | 0.041 | 0.082 |
| Does PPI improve the quality of your research? | 5.704 | 1.284 | 31.433 | 0.029 | 0.061 |
| Do PPI reduce and/or harm the quality of your research? | 0.819 | 0.243 | 2.725 | 0.743 | 0.743 |
| How often do you include PPI in your more recent research projects and/or articles? | - | - | - | - | - |

* Adjusted by region

** Were excluded the responses "I prefer not to answer" in all models

Little familiar or not familiar taken as reference variable

'- Convergency problems related to the lack of observations in the category “Little familiar or not familiar” in the dependent variable
